# Essential Oils from Mediterranean Plants Inhibit In Vitro Monocyte Adhesion to Endothelial Cells from Umbilical Cords of Females with Gestational Diabetes Mellitus

**DOI:** 10.3390/ijms24087225

**Published:** 2023-04-13

**Authors:** Valeria Schiavone, Tea Romasco, Nadia Di Pietrantonio, Stefania Garzoli, Carola Palmerini, Pamela Di Tomo, Caterina Pipino, Domitilla Mandatori, Rossella Fioravanti, Elena Butturini, Manuela Sabatino, Maria Pompea Antonia Baldassarre, Rino Ragno, Assunta Pandolfi, Natalia Di Pietro

**Affiliations:** 1 Department of Medical, Oral and Biotechnological Sciences, “G. d’Annunzio” University of Chieti-Pescara, 66100 Chieti, Italy; 2 Center for Advanced Studies and Technology—CAST, “G. d’Annunzio” University of Chieti-Pescara, 66100 Chieti, Italy; 3 Department of Pharmaceutical Chemistry and Technology, Sapienza University of Rome, 00185 Roma, Italy; 4 Department of Neurosciences, Biomedicine and Movement Sciences, Section of Biochemistry, University of Verona, 37129 Verona, Italy; 5 Rome Center for Molecular Design-RCMD, Department of Pharmaceutical Chemistry and Technology, Sapienza University of Rome, 00185 Roma, Italy; 6 Department of Medicine and Aging Sciences, “G. d’Annunzio” University Chieti-Pescara, 66100 Chieti, Italy

**Keywords:** essential oils, anise, laurel, anti-inflammatory, HUVEC, VCAM-1, inflammation, diabetes, NF-κB p65, endothelial dysfunction

## Abstract

Essential oils (EOs) are mixtures of volatile compounds belonging to several chemical classes derived from aromatic plants using different distillation techniques. Recent studies suggest that the consumption of Mediterranean plants, such as anise and laurel, contributes to improving the lipid and glycemic profile of patients with diabetes mellitus (DM). Hence, the aim of the present study was to investigate the potential anti-inflammatory effect of anise and laurel EOs (AEO and LEO) on endothelial cells isolated from the umbilical cord vein of females with gestational diabetes mellitus (GDM-HUVEC), which is a suitable in vitro model to reproduce the pro-inflammatory phenotype of a diabetic endothelium. For this purpose, the Gas Chromatographic/Mass Spectrometric (GC-MS) chemical profiles of AEO and LEO were first analyzed. Thus, GDM-HUVEC and related controls (C-HUVEC) were pre-treated for 24 h with AEO and LEO at 0.025% *v*/*v*, a concentration chosen among others (cell viability by MTT assay), and then stimulated with TNF-α (1 ng/mL). From the GC-MS analysis, trans-anethole (88.5%) and 1,8-cineole (53.9%) resulted as the major components of AEO and LEO, respectively. The results in C- and GDM-HUVEC showed that the treatment with both EOs significantly reduced: (i) the adhesion of the U937 monocyte to HUVEC; (ii) vascular adhesion molecule-1 (VCAM-1) protein and gene expression; (iii) Nuclear Factor-kappa B (NF-κB) p65 nuclear translocation. Taken together, these data suggest the anti-inflammatory efficacy of AEO and LEO in our in vitro model and lay the groundwork for further preclinical and clinical studies to study their potential use as supplements to mitigate vascular endothelial dysfunction associated with DM.

## 1. Introduction

Essential oils (EOs) are heterogeneous mixtures of numerous volatile compounds produced by plants’ secondary metabolism able to capture the plants’ scent and flavor [1]. Monoterpenes, sesquiterpenes, and related oxygenated derivatives are the main components of EOs, which provide them with biological properties and the characteristic essence. In addition to these, EOs also consist of a minor amount of aliphatic and aromatic compounds derived from phenylpropane, all characterized by a low molecular weight. It is important to underline that, as reported by the literature, the final chemical composition also depends on the extraction methods. In this regard, EOs can be obtained through distillation (via steam and/or water) or mechanical methods, such as cold pressing [2]. In any case, the bioactivities of a particular EO are mostly related to the synergistic effects of several chemical constituents, while no individual compounds show bioactivity [3].

Much evidence from in vitro and animal studies proves that EOs have multiple biological activities, such as anticancer [4,5], antiviral [6,7,8,9], antibacterial [10,11,12,13], antifungal [14,15], antibiofilm [16,17,18], anti-inflammatory [19,20,21], and antioxidant [19,22,23,24,25] effects, which constitute the starting point for the in vivo assessment of those properties in various therapeutic and cosmetic fields [26,27,28,29,30,31,32,33]. Nevertheless, since great consideration has been given to the effective use of EOs in clinical procedures, further systematic animal studies and clinical investigations are needed. In recent years there has been a growing interest in the study of EOs for their potential beneficial effects on diabetes mellitus (DM) and metabolic diseases related to obesity [3,34,35,36].

As a matter of fact, it has been observed that the possible use of EOs and some of their isolated constituents, such as monoterpenoids, improve and reduce the risk factors associated with cardiovascular diseases [37]. In particular, the study of Rajeshwari et al. [38] showed a significant reduction in fasting blood glucose levels following a daily treatment of 60 days with anise seeds powder in 20 patients with type 2 DM (in the age group of 40–60 years with no other specific complications). Interestingly, the antidiabetic, hypolipidemic, and antioxidant activities were a result of the synergistic action of antioxidants and phytochemicals (e.g., β-carotene, Vitamin A, C, and E) present in the anise seeds. In addition, other animal studies suggested that anise extracts could play an important role in blood glucose and type 2 DM management [39].

Along with anise, a recent study [40] showed that the leaf extract of another typical Mediterranean plant, *Laurel nobilis*, significantly decreased blood glucose levels and restored altered liver enzymes, urea, creatine kinase, total protein levels, calcium, and ferritin in six streptozotocin (STZ)-induced DM rats after a daily consumption period of 28 days when compared to the same number of untreated diabetic rats. Alam Khan et al. [41] have also proved that the encapsulation of 1, 2, or 3 g of ground bay leaves significantly reduces serum glucose, total cholesterol, Low-Density Lipoprotein (LDL) cholesterol, and triglycerides, and increases the High-Density Lipoprotein (HDL) cholesterol levels after 30 days in patients with type 2 DM with respect to a placebo group (a total of 40 individuals with more than 40 years were included and continued their normal diets and diabetic medications, except for insulin therapy, throughout the study). In relation to this, the positive effects of anise and laurel consumption on reducing the risk of cardiovascular disease were demonstrated, significantly improving the lipid profile with the increase of HDL cholesterol concentration and the decrease of LDL, not only in patients with type 2 DM but also in healthy people [41,42]. More specifically, in several in vitro and animal studies, it could be found that the phenolic acids and flavonoids contained in anise, such as *p*-coumaric acid, gallic aid, cinnamic acid, chlorogenic acid, and ferulic, besides catechin and rutin, contributed to prominently affecting the HDL lipid composition and to its atheroprotective action, exhibiting a radical scavenging activity, together with 1,8-cineol and other components (eugenol, acetyl and methyl eugenol, α- and β-pinene, phellandrene, linalool, geraniol, and terpineol) of laurel [43,44,45]. Unfortunately, nowadays most of the studies that can be found in the literature are based on cell culture or animal models rather than human studies, yet their effectiveness in humans is necessary to confirm through future research. Despite this, on this basis, it is possible to consider that both anise and laurel EOs (AEO and LEO, respectively) may serve as support for the management of type 2 DM and cardiovascular disease. However, to date, there is no evidence of their efficacy in improving or preventing endothelial dysfunction, which is considered one of the first steps in the atherosclerotic process associated with DM.

In this regard, in presence of persistent hyperglycemia, the activity of nitric oxide (NO) in preventing leukocyte adhesion and maintaining the endothelium in an anti-inflammatory state is compromised and the transcription factor Nuclear Factor-kappa B (NF-κB) p65 signaling pathway is activated [46,47,48,49]. In turn, this latter induces the expression of cytokines, chemokines, and adhesion molecules, such as Vascular Cell Adhesion Molecule-1 (VCAM-1), Intercellular Adhesion Molecule-1 (ICAM-1), and E-selectin, which participate in the recruitment of monocytes to the endothelial surface, playing a key role in the onset of inflammation and endothelial dysfunction, as well as in the progression of diabetic vascular complications [50,51].

The mechanisms determining the development of endothelial dysfunction are complex and not limited only to hyperglycemia. For example, during pregnancies complicated by diabetes and/or obesity, although also present in physiologically normal pregnancies, an increase of circulating inflammatory molecules and reduced levels of anti-inflammatory molecules, such as IL-10 and adiponectin, were demonstrated [52]. Gestational diabetes mellitus (GDM) later leads to an imbalance between pro- and anti-inflammatory/oxidative molecules that may support the development of early vascular senescence and endothelial dysfunction in offspring [53]. In general, the chronic state of low-grade inflammation caused by obesity is known to interfere with the normal transmission of insulin signaling, favoring the onset of type 2 DM and further propagating the state of chronic inflammation and vascular dysfunction even years before the diagnosis of diabetes [34,54]. Based on the above premises, the present study was undertaken to investigate the potential anti-inflammatory role of AEO and LEO on primary endothelial cell cultures isolated from the umbilical cord vein of females with gestational diabetes mellitus (GDM-HUVEC), which represent a valuable in vitro model for reproducing the in vivo pro-inflammatory vascular phenotype associated with chronic hyperglycemia [55]. In particular, the use of AEO and LEO is proposed to contribute to decrease the burden of inflammation in vascular cells by targeting the earlier altered mechanisms involved in the onset of endothelial dysfunction.

## 2. Results

### 2.1. Chemical Composition of EOs

The chemical characterization of AEO and LEO was performed by Gas Chromatographic/Mass Spectrometric (GC-MS) analysis. In total, 22 components were identified and listed in Table 1. The monoterpene content found in LEO (93.9%) is markedly higher than that found in AEO (3.8%), where trans-anethole (88.5%) was the most abundant component followed by estragole (3.8%) and *p*-anisaldheyde (2.1%). On the other side, 1,8-cineole (53.9%) and *α*-terpinyl acetate (18.8%) were the molecules with higher percentage mean values detected in LEO.

### 2.2. Effects of AEO and LEO on Cell Viability

**Figure 1:**
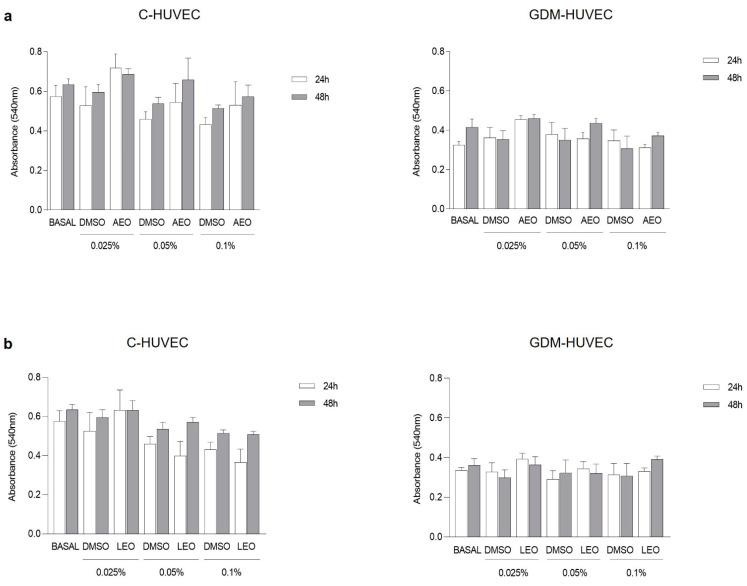
C- and GDM-HUVEC cell viability after 24 h and 48 h of treatment with (**a**) AEO and (**b**) LEO at different concentrations (0.025, 0.05, and 0.1% *v*/*v*) or with medium alone (BASAL). Results are shown as mean ± Standard Error of the Mean (SEM). All experiments were conducted in technical and biological triplicate using at least 3 different primary culture strains of C- and GDM-HUVEC.

First, the potential cytotoxic effect of EOs derived from anise and laurel plants, both widely spread throughout the Mediterranean basin, was evaluated. For this purpose, the 3-(4,5-dimethylthiazolyl-2)-2, 5-diphenyltetrazolium bromide (MTT) cell viability assay was performed on GDM-HUVEC and related controls (C-HUVEC) treated with three different concentrations of AEO and LEO (0.025, 0.05, and 0.1% *v*/*v*) for 24 h and 48 h. The obtained results showed that C- and GDM-HUVEC viability remained stable and was not altered by treatment with AEO and LEO at all concentrations tested (Figure 1). 

Moreover, the graphics showed no statistically significant differences between cells treated with different concentrations of EOs diluted in Dimethyl Sulfoxide (DMSO) and the corresponding concentrations of DMSO diluted in the cell culture medium.

Based on these results, it was decided to select 0.025% *v*/*v* as the concentration of AEO and LEO to be tested at 24 h for their potential anti-inflammatory role in the subsequent experiments. In addition, since DMSO alone did not exert a cytotoxic effect at any concentrations or time points, the following experiments were conducted excluding this experimental condition.

### 2.3. Effects of AEO and LEO on U937 Monocyte–HUVEC Interaction

**Figure 2:**
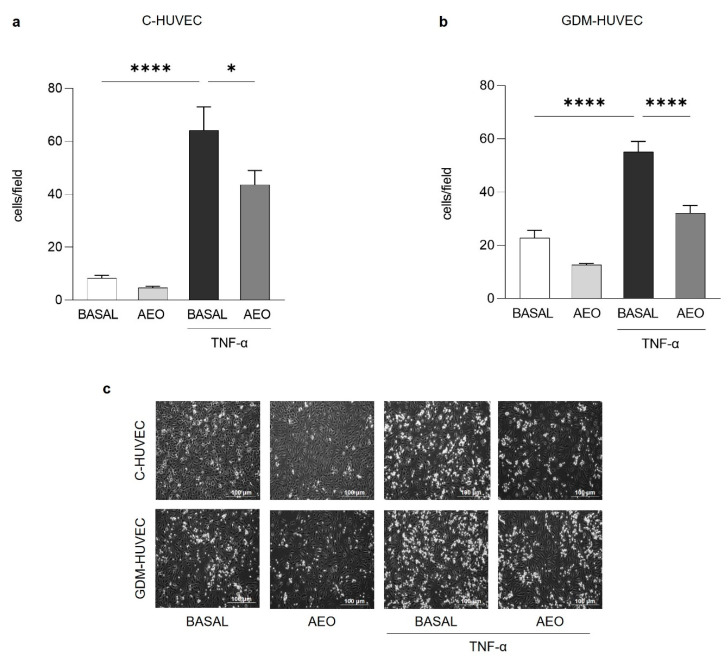
Effects of AEO on U937 monocyte adhesion to C- and GDM-HUVEC. Cells were serum-starved and incubated for 16h with TNF-α (1 ng/mL) following 24 h of pre-incubation with AEO (0.025% *v*/*v*) or with medium alone (BASAL). Quantitative data express the number of U937 cells adhering within a high-power field (3.5 mm^2^). Each measurement is expressed as mean ± SEM of adhering cells from 3 independent experiments, consisting of 8 counts per condition. (**a**) **** *p* < 0.0001 TNF-α vs. BASAL; * *p* < 0.05 TNF-α vs. TNF-α + AEO. (**b**) **** *p* < 0.0001 TNF-α vs BASAL; **** *p* < 0.0001 TNF-α vs. TNF-α+AEO. (**c**) Representative images of U937 cell adhesion to C- and GDM-HUVEC. Scale bar: 100 µm.

The monocyte-adhesion assay was performed to assess the in vitro potential anti-inflammatory role of AEO and LEO on endothelial cells stimulated or not with Tumoral Necrosis Factor-α inflammatory cytokine (TNF-α, 1 ng/mL), which is useful to mimic the in vivo inflammatory condition. As shown in Figure 2, the pre-treatment with AEO (0.025% *v*/*v*) for 24 h significantly reduced the TNF-α stimulated U937 monocyte adhesion to both C- and GDM-HUVEC, while in basal conditions, this effect never reached statistically significant values.

**Figure 3:**
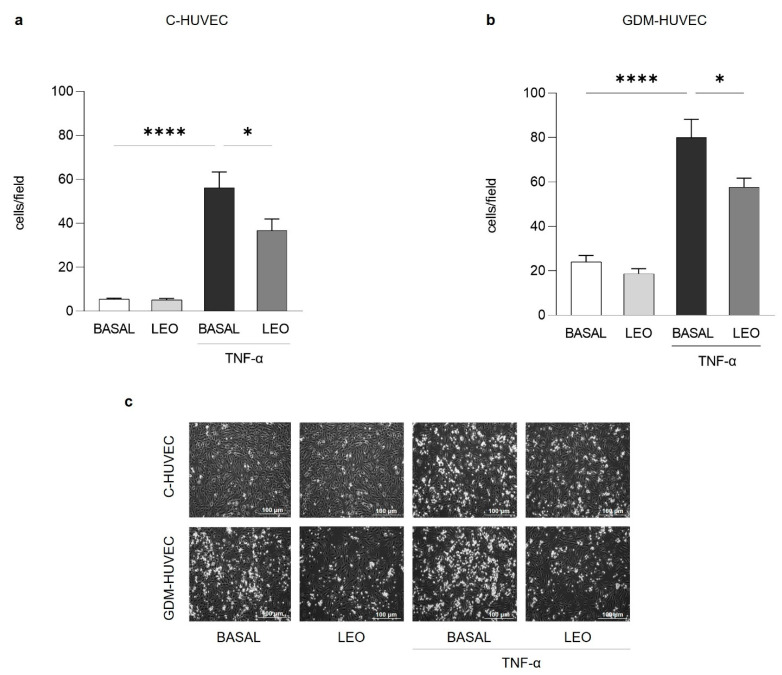
Effects of LEO on U937 monocyte adhesion to C- and GDM-HUVEC. Cells were serum-starved and incubated for 16h with TNF-α (1 ng/mL) following 24 h of pre-incubation with LEO (0.025% *v*/*v*) or with medium alone (BASAL). Quantitative data express the number of U937 cells adhering within a high-power field (3.5 mm^2^). Each measurement is expressed as mean ± SEM of adhering cells from 3 independent experiments, consisting of 8 counts per condition. (**a**,**b**) **** *p* < 0.0001 TNF-α vs. BASAL; * *p* < 0.05 TNF-α vs. TNF-α+LEO. (**c**) Representative images of U937 cell adhesion to C- and GDM-HUVEC. Scale bar: 100 µm.

Similarly, the treatment with the same concentration of LEO for 24 h significantly decreased U937 cell adhesion to TNF-α stimulated C- and GDM-HUVEC (Figure 3).

### 2.4. Effects of AEO and LEO on VCAM-1 Membrane Exposure Levels and mRNA Expression

On the basis of the results obtained from the U937 monocyte adhesion assay and since it is well known that the vascular adhesion molecules participate in the recruitment of monocytes into the subendothelium [51], the effect of AEO and LEO on VCAM-1 membrane exposure levels was then evaluated by flow cytometry analysis. 

**Figure 4:**
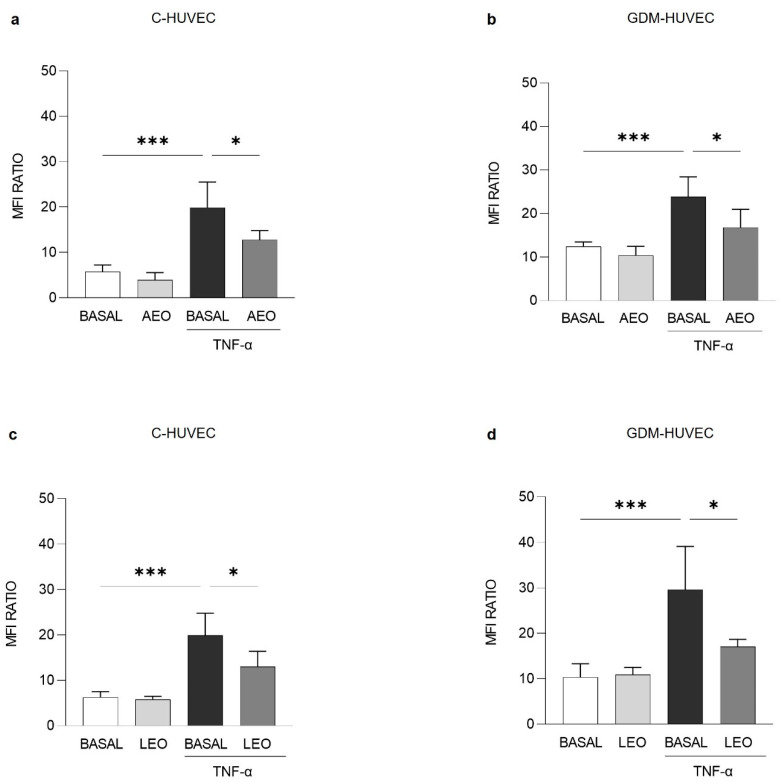
Effects of (**a**,**b**) AEO and (**c**,**d**) LEO on VCAM-1 membrane exposure levels in C- and GDM-HUVEC. Cells were serum-starved and incubated for 16h with TNF-α (1 ng/mL), following 24 h of pre-incubation with AEO or LEO (0.025% *v*/*v*) or with medium alone (BASAL). Results are presented as Mean Fluorescence Intensity (MFI) Ratio and shown as mean ± Standard Deviation (SD). All experiments were conducted in technical and biological triplicate using at least 3 different primary culture strains of C- and GDM-HUVEC. (**a**–**d**) *** *p* < 0.001 TNF-α vs. BASAL; * *p* < 0.05 TNF-α vs AEO+TNF-α or LEO+TNF-α.

After pre-incubating TNF-α stimulated cells with AEO and LEO (0.025%) for 24 h, VCAM-1 membrane exposure levels were significantly reduced in both C- (Figure 4a and Figure 4c, respectively) and GDM-HUVEC (Figure 4b and Figure 4d, respectively).

**Figure 5:**
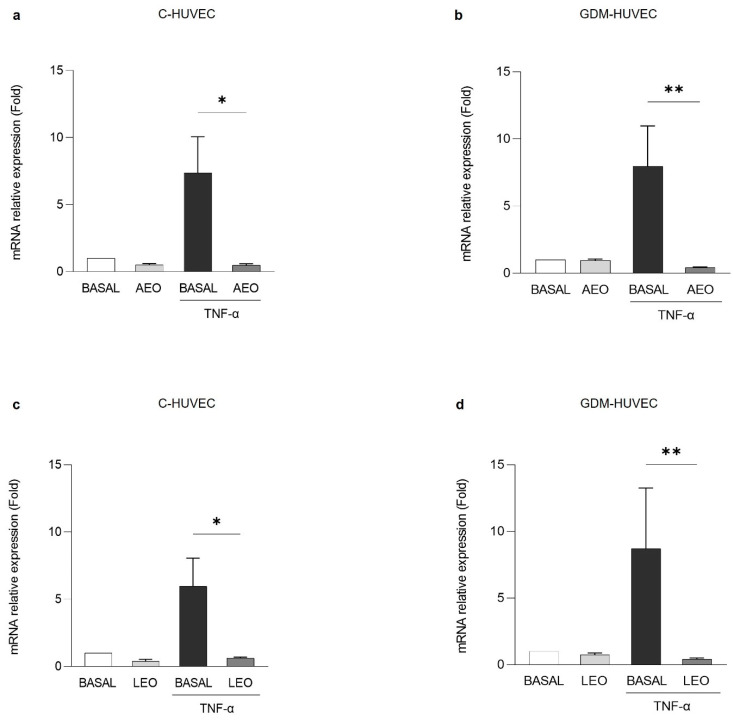
Effects of (**a**,**b**) AEO and (**c**,**d**) LEO on VCAM-1 mRNA expression in C- and GDM-HUVEC. Cells were serum-starved and incubated for 16 h with TNF-α (1 ng/mL), following 24 h of pre-incubation with AEO or LEO (0.025% *v*/*v*) or with medium alone (BASAL). Results of RT-qPCR analysis are shown as individual values and mean ± SD of at least 3 independent experiments. (**a**,**c**) * *p* < 0.05 TNF-α vs. AEO+TNF-α or LEO+TNF-α; (**b**,**d**) ** *p* < 0.01 TNF-α vs. AEO+TNF-α or LEO+TNF-α.

Then, as shown in Figure 5, VCAM-1 mRNA expression was evaluated by Quantitative Real-Time PCR (RT-qPCR). VCAM-1 mRNA expression levels resulted in significantly reduced TNF-α stimulated C- and GDM-HUVEC after pre-treatment with AEO (Figure 5a and Figure 5b, respectively) and LEO (Figure 5c and Figure 5d, respectively).

### 2.5. Effects of AEO and LEO on NF-κB p65 Nuclear Translocation

NF-κB p65 is a nuclear transcription factor, which plays a key role in the inflammatory response [56]. In particular, the activation of the NF-κB signaling pathway, which occurs through its nuclear translocation, can promote the expression of vascular adhesion molecules [57]. Hence, in order to mechanistically support the potential anti-inflammatory role of AEO and LEO in C- and GDM-HUVEC, the NF-κB p65 activation was investigated by immunofluorescence staining.

**Figure 6:**
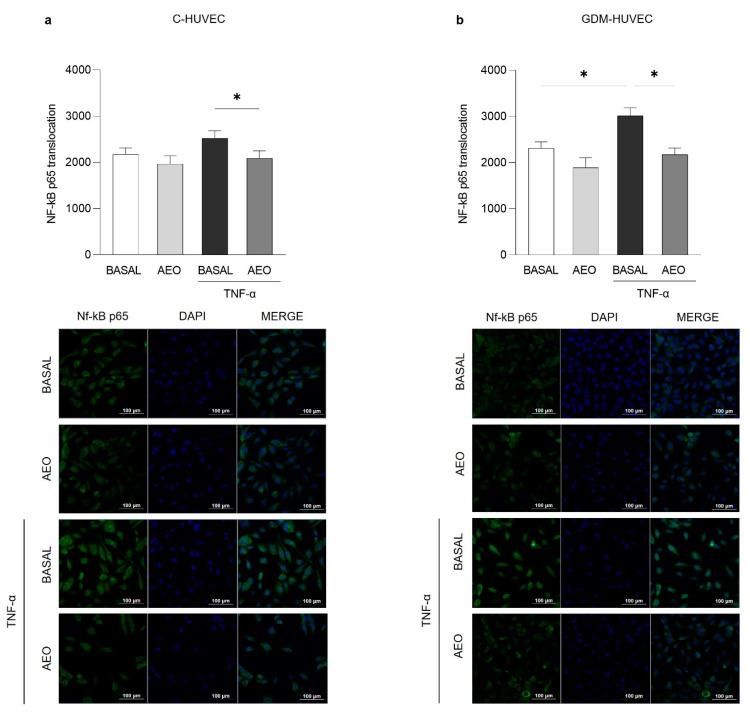
Effects of AEO on NF-κB p65 nuclear translocation in C- and GDM-HUVEC. Graphs and representative immunofluorescence images showed NF-κB p65 nuclear translocation in C- and GDM-HUVEC by immunofluorescence staining. Cells have been marked with NF-κB p65 primary antibody and Alexa Fluor 488 anti-rabbit secondary antibody, and nuclei were stained with 40, 6-diamidino-2-phenylindole (DAPI) following 24 h of treatment with AEO (0.025%). Data, calculated as the ratio of the MFI of NF-κB p65 (green) on the MFI of nuclei (blue), were obtained by analyzing at least 3 different fields for each image with ImageJ software (NIH, USA ImageJ software, public domain available at: http://rsb.info.nih.gov/nih-image/). Results are expressed as mean ± SEM. (**a**,**b**) * *p* < 0.05 TNF-α vs. AEO+TNF-α; (**b**) * *p* < 0.05 TNF-α vs. BASAL. Scale bar: 100 µm.

**Figure 7:**
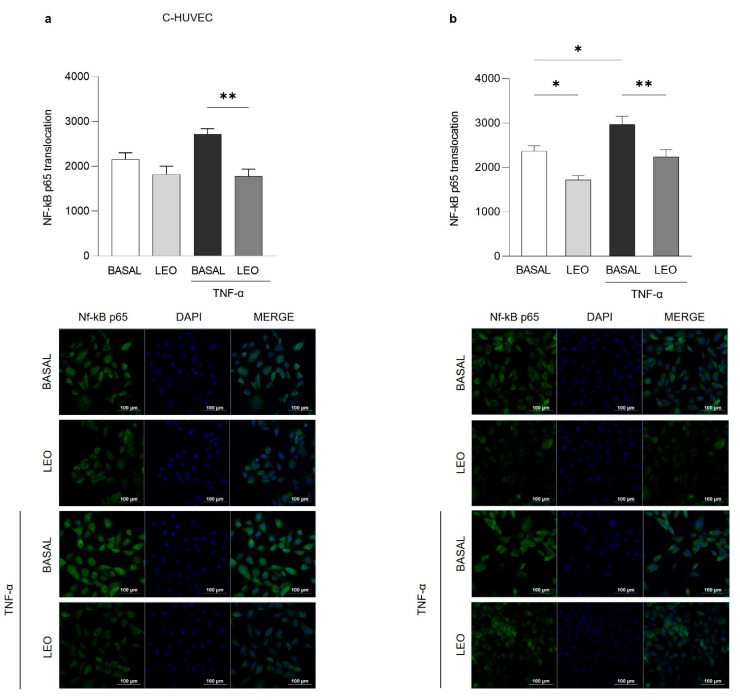
Effects of LEO on NF-κB p65 nuclear translocation in C- and GDM-HUVEC. Graphs and representative immunofluorescence images show NF-κB p65 nuclear translocation in C- and GDM-HUVEC by immunofluorescence staining. Cells have been marked with NF-κB p65 primary antibody and Alexa Fluor 488 anti-rabbit secondary antibody, and nuclei were stained with DAPI, following 24 h of treatment with LEO (0.025%). Data, calculated as the ratio of the MFI of NF-κB p65 (green) on the MFI of nuclei (blue), were obtained by analyzing at least 3 different fields for each image with ImageJ software (NIH, ImageJ software). (**a**,**b**) ** *p* < 0.01 TNF-α vs. LEO+TNF-α; (**b**) * *p* < 0.05 BASAL vs. TNF-α or LEO. Scale bar: 100 µm.

As shown in Figure 6 and Figure 7, both EOs (0.025% *v*/*v*) were able to significantly reduce TNF-α-induced NF-κB p65 cytoplasm–nucleus translocation in both C- and GDM-HUVEC, while in basal conditions, this effect reached statistical significance only in GDM-HUVEC pre-incubated with LEO (Figure 7b).

## 3. Discussion

Endothelial dysfunction, associated with an increased oxidative stress and inflammatory condition, represents the first key event in the onset of vascular complications in DM [58]. Among natural bioactive compounds, the use of EOs in the moderation of inflammatory disorders nowadays represents an increasing research field [59]. In fact, EOs are showing growing interest in food and cosmetic industries, as well as in the human health field [1,26,27,28,29,30,31,32].

This study was based on the fact that the EOs and some of their active components have been shown to potentially exert several biological activities including modulation of various cardiovascular risk factors, such as oxidative stress and inflammation [37]. In particular, the in vitro use of EOs is here proposed to mitigate the burden of inflammation in vascular cells by targeting the earlier altered mechanisms involved in the onset of endothelial dysfunction.

Hence, we investigated the potential anti-inflammatory effects of AEO and LEO, which are particularly abundant in Mediterranean aromatic plants, by using GDM-HUVEC, a valuable cellular model to study the early triggering events of vascular complications associated with DM [55,60].

To this end, the potential cytotoxic effect of different concentrations of AEO and LEO on C- and GDM-HUVEC was first evaluated, demonstrating that they did not affect cell viability. Especially, the concentration of 0.025% *v*/*v* showed better results in terms of vitality in comparison with other concentrations used and baseline. On this basis, it was decided to perform all the subsequent tests with the use of AEO and LEO at a concentration of 0.025% *v*/*v* for 24 h.

Then, to investigate the possible AEO and LEO anti-inflammatory activity in vitro, the U937 monocyte–endothelial cell interaction rate was determined, since it is considered one of the earliest events involved in atherosclerosis [61]. After attaching to the endothelium, monocytes subsequently invade the vascular wall, where they play a central role in triggering inflammation [46,51]. Here, it was found that the stimulation with a low concentration of TNF-α induced an increase in the U937 monocyte–cell interaction in both cell populations. The pre-treatment with AEO, as well as LEO, significantly reduced the number of adhered U937 cells in both C- and GDM-HUVEC, even if AEO determined a stronger decrease in U937 adhesion to GDM-HUVEC with respect to LEO. On the other hand, a not significant decrease could be detected in basal conditions for both cell populations after treatment with AEO and LEO.

To further investigate this process, since it is well known that vascular adhesion molecules, such as VCAM-1, participate in the recruitment of monocytes to the endothelium [55,62,63], the effect of AEO and LEO on VCAM-1 membrane exposure levels in vitro was evaluated by flow cytometry analysis. After pre-incubating TNF-α stimulated cells with AEO and LEO for 24 h, VCAM-1 total expression and membrane exposure levels were significantly reduced in both C- and GDM-HUVEC. 

Although not only VCAM-1, but also other vascular adhesion molecules, such as ICAM-1 or E-selectin, are known to be responsible for U937 cell adhesion to HUVEC [64], these data have a prominent role, considering that increased interest in the possible consumption of these compounds as support for the modulation of vascular diseases is growing. Indeed, several dietary bioactive components, although not considered essential, may hold important benefits for human health, being nowadays widely used by the food industry [34,35,38,65,66]. The efficacy of the processed product for pharmaceutical, cosmeceutical, and nutraceutical applications is directly linked to WHO guidelines on good agricultural and collection practices (WHO-GACP) and the good herbal processing (GHPP) practices, which are helpful in further enhancing the therapeutic efficacy, safety, and potential of the processed products [67,68].

Furthermore, it is well known that NF-κB activation can induce the transcription of a large number of genes implicated in vascular inflammation, including cytokines, chemokines, and adhesion molecules [57]. As a matter of fact, several studies performed on cells, laboratory animals, and humans have demonstrated their implication in endothelial dysfunction and atherosclerosis [69,70,71], both of which phenomena could be modulated by using different pharmacological or natural approaches [56,72,73,74]. Hence, to further investigate the potential anti-inflammatory effect of AEO and LEO on C- and GDM-HUVEC, NF-κB p65 activation was investigated. Interestingly, both EOs were able to significantly reduce TNF-α-induced NF-κB p65 cytoplasm–nucleus translocation in both C- and GDM-HUVEC, thus confirming the capability of AEO and LEO in reducing vascular inflammation in vitro. Of note, NF-κB p65 nuclear translocation reached also statistical significance in GDM-HUVEC pre-incubated with LEO in basal conditions.

Nevertheless, several cellular studies have reported the capability of other EOs in alleviating inflammation [19,20,56,65,75], but to the best of our knowledge, this is the first report demonstrating the anti-inflammatory role of AEO and LEO in a GDM-HUVEC in vitro model.

Collectively, these findings lay the foundations for further studies in demonstrating that these EOs may help to protect human vessel endothelium from damages related to chronic hyperglycemia and type 2 DM by interfering with signal mechanisms related to vascular inflammation. These results are in line with previous in vitro and in vivo evidence based on the anti-inflammatory properties expressed by other natural bioactive compounds on HUVEC [65,75,76] and may explain some of the beneficial effects reported by the implementation of the polyphenol-rich Mediterranean diet and its related bioactive molecules [77,78,79,80].

In conclusion, the following findings reported by AEO and LEO properties in this GDM in vitro model provided evidence that these molecules may act on the vascular inflammatory state by decreasing U937 monocyte adhesion thanks to the regulation of the NF-κB p65 signaling pathway. This may represent another interesting mechanism to further elucidate the possible beneficial role of AEO and LEO in mitigating endothelial dysfunction.

Despite important features of these extracts that need to be improved, such as their high volatility and hydrophobicity, which could compromise their bioavailability, the information being generated by in vitro assays obviously needs to be confirmed through systematic animal studies and clinical investigations. With the help of further research, the EOs of anise and laurel could find large applications in the healthcare and nutraceutical fields, since endothelial dysfunction represents a common triggering factor of several vascular diseases.

Further studies are also required in due course on a larger number of other EOs with the purpose of establishing some quantitative composition–activity relationships (QCAR) to dissect the chemical components mainly responsible for the herein-reported biological effects.

## 4. Materials and Methods

### 4.1. Chemicals

DMSO (CAT. 102950) was purchased from Sigma-Aldrich (Merk Life Science S.r.l., Milan, Italy). TRIzol^TM^ reagent (CAT. 15596026), High-Capacity cDNA Reverse Transcription Kit (CAT. 4368813), TaqMan Gene Expression Assays probes, and TaqManUniversal Master mix II (CAT. 4440038) were purchased from Thermo Fisher Scientific (Waltham, MA, USA).

### 4.2. GC/MS Analysis

AEO and LEO were subjected to GC/MS analysis to characterize their composition, as previously described [81]. Briefly, a Stabilwax fused-silica capillary column (Restek, Bellefonte, PA, USA) (60 m × 0.25 mm, 0.25 mm film thickness) was used and the GC oven program was as follows: isothermal exposure at 60 °C for 5 min, which was then ramped to 220 °C at a rate of 6 °C min^−1^_,_ and finally, isothermal exposure at 220 °C for 20 min. The identification of components was performed by matching their mass spectra with those stored in the Wiley and NIST 02 mass spectra library databases. Furthermore, the linear retention indices (LRIs) (relative to C_8_–C_30_ aliphatic hydrocarbons) were calculated and compared with available retention data presented in the literature (Nist) [82]. Relative percentages of all identified components were obtained by peak area normalization from Gas Chromatography–Flame Ionization Detection (GC-FID) chromatograms without the use of an internal standard or correction factors and expressed in percentages [10]. All analyses were repeated twice.

### 4.3. EOs Dilution

EOs (Farmalabor srl, Assago, Italy) were dissolved in DMSO at 50 mg/mL to obtain complete solubilization, and further diluted in the medium for cell culture experiments, always resulting in a DMSO concentration that has no effect on cell viability.

### 4.4. Cell Cultures and Experimental Protocols

Umbilical cords were collected at full-term delivery from healthy Caucasian mothers (Control, C) and those with GDM, randomly selected at Pescara Hospital (Pescara, Italy). All procedures were performed in agreement with the ethical standards of the Institutional Committee on Human Experimentation (Reference Number: 1879/09COET) and with the Declaration of Helsinki Principles. Informed consent was signed by each participating subject after approval of the protocol by the Institutional Review Board.

Primary C- and GDM-HUVEC were obtained, cultured (5% CO_2_ and 37 °C), and used between the 3rd and 5th passages in vitro, as previously described [83]. 

After that, HUVEC were grown to sub-confluence in complete Cell Culture Media–Dulbecco’s Modification of Eagle’s Medium (DMEM, CAT. 10014CV, Corning^TM^, Glendale, AZ, USA), M199 with Earle’s salts and L-glutamine (CAT. 10060CV, Corning^TM^, Glendale, AZ, USA), L-glutamine (CAT. 25005CL, Corning^TM^, Glendale, AZ, USA), Penicillin-Streptomycin (CAT. P0607100, Corning^TM^, Glendale, AZ, USA), and Fetal Bovine Serum (FBS, CAT. 41A0045K, Gibco-Life Technologies, Thermo Fisher Scientific, Waltham, MA, USA) for all the following experimental tests.

First of all, the potential cytotoxic effect of AEO and LEO, extracted from the related Mediterranean plants, was evaluated in order to find the most suitable EO concentration to perform all the subsequent experiments. For this aim, C- and GDM-HUVEC were stimulated for 24 h and 48 h with different concentrations of oils (0.025%, 0.05%, and 0.1% *v*/*v*) dissolved in DMSO. Then, cell viability was evaluated.

The obtained results allowed us to select the best AEO and LEO concentrations (0.025% *v*/*v*) to test for their potential anti-inflammatory role. For this purpose, in each experiment, C- and GDM-HUVEC were serum-starved (with 0.1% FBS) and incubated for 16h with TNF-α (1 ng/mL, CAT. PHC3015L, Gibco-Life Technologies, Thermo Fisher Scientific, Waltham, MA, USA), following 24 h of pre-incubation with AEO and LEO, or with medium alone as a basal condition.

### 4.5. MTT Assay

The effect of AEO and LEO on C- and GDM-HUVEC viability was assessed by the MTT (Sigma-Aldrich, Merk Life Science S.r.l., Milan, Italy) assay according to the manufacturer’s instructions.

Cells were seeded into 96-well plates, grown to confluence, and stimulated for 24 h and 48 h with different EO concentrations dissolved in DMSO: 0.025%, 0.05%, and 0.1% *v*/*v*, or with DMSO and the medium alone as controls. Briefly, after 24 h and 48 h of treatment, a solution of diluted MTT in phosphate buffer (PBS; Sigma Aldrich, Merk Life Science S.r.l., Milan, Italy) was added to each well at a concentration of 0.5 mg/mL. Following 3 h of incubation at 37 °C, 0.2 mL of DMSO per well was added for 30 min. Then, cell viability was evaluated by measuring the spectrometric absorbance (ABS) at a 540 nm wavelength using a microplate reader (SpectraMAX 190, Molecular Devices Inc., Sunnyvale, CA, USA).

### 4.6. U937 Monocyte Adhesion Assay

The adhesion of the human monocyte line U937 to C- and GDM-HUVEC was evaluated in a basal state, after 16h of TNF- α (1 ng/mL) exposure, and in presence of AEO and LEO (after 24 h of pre-incubation) alone or in combination with TNF-α, as described in Section 4.4. U937 monocyte cell suspensions were added to each HUVEC monolayer under rotating conditions at room temperature (RT) and the assay was performed as previously described [76].

### 4.7. Flow Cytometry Analysis

The levels of VCAM-1 protein membrane exposure were evaluated using the flow cytometry analysis.

Briefly, cells were detached by 0.5% trypsin/0.2% EDTA solution (CAT. 59418C, Sigma-Aldrich, Milan, Italy), collected with PBS, and resuspended in Bovine Serum Albumin (BSA 0.5%, CAT. PR23225, Euroclone, Milan, Italy). Then, they were centrifugated at 800 rpm for 15 min at 4 °C. After that, cells were incubated with PE-labelled anti-VCAM-1 antibody (1:20, CAT. 305806, BioLegend, San Diego, CA, USA) for 30 min at RT.

All data were analyzed using FACS Diva (BD Bioscences, San Diego, CA, USA) and FlowJo v.8.8.6 software (TreeStar, Ashland, OR, USA) and expressed as MFI Ratio. The MFI Ratio was calculated by dividing the MFI of positive events by the MFI of negative events (MFI of the secondary antibody).

### 4.8. RTq-PCR

The total RNA was isolated and extracted from HUVEC using the TRIzol^TM^ reagent protocol, then the High-Capacity cDNA Reverse Transcription Kit was employed to synthesize cDNA.

The TaqMan Universal Master Mix II and TaqMan Gene Expression Assay probes for human VCAM-1 (Hs01003372_m1) and GAPDH (Hs02786624_g1) were used according to the manufacturer’s instructions. Gene expression was assessed with the ABI Prism 7900 Sequence Detection System (ThermoFisher Scientific, Waltham, MA, USA). The relative gene expression was calculated using the comparative 2^−∆∆CT^ method and results are expressed as the fold change related to the untreated control.

### 4.9. Nf-κB p65 Evaluation by Immunofluorescence

NF-κB p65 nuclear translocation in C- and GDM-HUVEC was assessed by immunofluorescence staining.

Cells were treated with AEO and LEO (0.025% *v*/*v*) for 24 h and then stimulated with TNF-α for 2 h to evaluate NF-κB cytoplasm–nucleus translocation. The assay was performed on C- and GDM-HUVEC seeded in a Chamber Polystyrene Vessel Tissue Culture Treated Glass Slide (BD Falcon, Franklin Lakes, NJ, USA) (30,000 cells/chamber). After 24 h of treatment, cells were fixed with 3% Paraformaldehyde (10 min at RT), permeabilized with a solution (HEPES 20 mM pH 7.4, saccharose 300 µM, NaCl 50 mM, MgCl_2_ 3 mM, and Triton X-100 0.5%), and incubated with NF-κB p65 primary antibody (1:50, overnight at 4 °C, CAT. P65C2284, Cell Signaling Technology, Milan, Italy) and Alexa Fluor 488 anti-rabbit secondary antibody (1:50, 30 min at RT, CAT. A11034, Invitrogen, Thermo Fisher Scientific, Waltham, MA, USA). Lastly, the nuclei were stained with DAPI (1:5000, 15 min at RT, CAT. D9542, Sigma-Aldrich, Merk Life Science S.r.l., Milan, Italy) and then observed using a confocal microscope (Zeiss LSM-800, Carl Zeiss Meditec AG, Oberkochen, Germany).

Data were expressed as the ratio of the MFI of NF-κB p65 (green) on the MFI of nuclei (blue) by analyzing at least 3 different fields for each image with ImageJ software (NIH, ImageJ software).

### 4.10. Statistical Analysis

All experimental data are presented as mean ± SD or SEM. All experiments were conducted in technical and biological triplicate using at least 3 different primary culture strains of C- and GDM-HUVEC.

Statistical analysis was performed using One-Way Analysis of Variance (ANOVA), Bonferroni multiple comparison test for post hoc comparison, and the Kruskal–Wallis or Dunn’s post hoc tests. *p*-values < 0.05 were considered statistically significant. Analysis and graphs were performed using GraphPad Prism Software Analysis (version 9).

## Figures and Tables

**Table 1:** AEO and LEO chemical composition.

N.	Component ^1^	LRI ^2^	LRI ^3^	AEO% ^4^	LEO% ^5^
1	*α*-pinene	1018	1021	0.3	3.2
2	*β*-pinene	1091	1099	-	2.3
3	sabinene	1110	1115	-	3.2
4	limonene	1198	1190	1.6	2.0
5	1,8-cineole	1201	1206	-	53.9
6	*γ*-terpinene	1240	1244	-	1.8
7	o-cymene	1279	1287	-	2.4
8	linalool	1545	1547	0.9	3.0
9	*α*-bergamotene	1581	1582	0.5	-
10	terpinen-4-ol	1606	1603	0.3	3.0
11	*β*-caryophyllene	1612	1619	0.4	-
12	estragole	1652	1655	3.8	-
13	*α*-terpinyl acetate	1685	1683	-	18.8
14	α-terpineol	1722	1719	0.2	-
15	myrtenol	1808	1804	-	0.3
16	trans-anethole	1840	1837	88.5	-
17	(*E*)-nerolidol	2025	2023	0.3	-
18	*p*-anisaldehyde	2030	2027	2.1	-
19	methyl eugenol	2036	2033	-	4.2
20	*p*-acetonylanisole	2172	2170	0.9	-
21	eugenol	2175	2172	-	1.9
22	isohomogenol	2191	2189	0.2	-
	SUM			100.0	100.0
	Monoterpenes			3.8	93.9
	Sesquiterpenes			0.7	-
	Other			95.5	6.1

^1^ The components are reported according to their elution order on Stabilwax polar column; ^2^ linear retention indices (LRI) measured on a polar column; ^3^ LRI from the literature (Nist); ^4^ percentage mean values of AEO components (%); ^5^ percentage mean values of LEO components; * LRI not available; - Not detected.

## Data Availability

Not applicable.
